# Bisphenol A and Hormone-Associated Cancers: Current Progress and Perspectives

**DOI:** 10.1097/MD.0000000000000211

**Published:** 2015-01-09

**Authors:** Hui Gao, Bao-Jun Yang, Nan Li, Li-Min Feng, Xiao-Yu Shi, Wei-Hong Zhao, Si-Jin Liu

**Affiliations:** From the Department of Obstetrics & Gynecology (HG, B-JY, LMF, X-YS, W-HZ), Beijing TianTan Hospital, Capital Medical University, Beijing 100050, China; Department of Gynecology (NL), Cancer Institute and Hospital, Chinese Academy of Medical Sciences (CAMS), Beijing 100021, China; State Key Laboratory of Environmental Chemistry and Ecotoxicology (S-JL), Research Center for Eco-Environmental Sciences, Chinese Academy of Sciences, Beijing 100085, China

## Abstract

Bisphenol A (BPA), a carbon-based synthetic compound, exhibits hormone-like properties and is present ubiquitously in the environment and in human tissues due to its widespread use and biological accumulation. BPA can mimic estrogen to interact with estrogen receptors α and β, leading to changes in cell proliferation, apoptosis, or migration and thereby, contributing to cancer development and progression. At the genetic level, BPA has been shown to be involved in multiple oncogenic signaling pathways, such as the STAT3, MAPK, and PI3K/AKT pathways. Moreover, BPA may also interact with other steroid receptors (such as androgen receptor) and plays a role in prostate cancer development. This review summarizes the current literature regarding human exposure to BPA, the endocrine-disrupting effects of BPA, and the role of BPA in hormone-associated cancers of the breast, ovary, and prostate.

## INTRODUCTION

Bisphenol A (BPA) is an industrial synthetic chemical that is used to make certain plastics and epoxy resins and has been commercially available since 1957. Currently, BPA is one of the highest volume chemicals produced worldwide (more than 6 × 10^9^ lb/year). BPA is widely present in many hard plastic bottles and metal-based food and beverage cans. Although BPA is a colorless solid that is soluble in organic solvents and poorly soluble in water, human exposure does occur when BPA leaches from plastic-lined food and beverage cans, water bottles, and some dental sealants.^1,2^ BPA leaching occurs when plastic and epoxy resin-containing bottles and cans are heated or repeatedly washed. Thus, BPA is ubiquitously found in the environment throughout the world.^3,4^

BPA has been the focus of widespread concern due to the fact that it interferes with endocrine signaling pathways even at extremely low doses. BPA is a diphenyl compound that contains two hydroxyl groups in the “para” position, making it remarkably similar to the synthetic estrogen, diethylstilbestrol. Particularly, many studies have shown that BPA can activate estrogen receptors (ERs) α and β.^5,6^ To date, multiple lines of evidence have indicated that BPA has estrogen-like activity and exhibits developmental toxicity in the reproductive organs and inhibitory effects on testosterone synthesis.^7–9^ Thus, in vitro and in vivo studies have revealed links between BPA exposure and hormone-related cancers, including breast, prostate, and ovarian cancers and endometrial carcinoma. In this review, we summarize the current literature regarding human exposure to BPA and the role of BPA in the development of hormone-related cancers.

## HUMAN EXPOSURE TO BPA AND ITS METABOLISM

BPA can leach into food and beverages through the daily use of tin cans, baby bottles, reusable plastic water bottles, and polycarbonate plastic containers. The rate of BPA leaching increases when polycarbonate is scratched or discolored.^10,11^ Heat and non-neutral pH conditions (either acidic or basic) are two factors that influence BPA release, because hydrolysis of the ester bond linking BPA monomers occurs with changes in temperature and pH,^12,13^ such as those that take place when BPA-containing plastics are cleaned with harsh detergents or contain acidic or high-temperature liquids. In a study evaluating BPA exposure in male workers who spray epoxy resin, the concentration of BPA in urine was found to be higher in the epoxy resin sprayers (median 1.06 μmol/mol creatinine) compared to the controls (median 0.52 μmol/mol creatinine) and the level of follicle-stimulating hormone (FSH) was also correlated with urinary BPA.^14^ Using different measurement techniques, BPA has been found to be present in human serum, urine, amniotic fluid, and breast milk in the populations of industrialized countries worldwide.^15^ In a reference population of 394 adults in the United States, BPA was detected in 95% of urine samples with a median concentration of 1.28 μg/L.^16^ The BPA concentration in human serum ranges from 0.2–1.6 ng/mL (0.88–7.0 nM).^17,18^ The U.S. Environmental Protection Agency has defined a reference dose of BPA to be 50 mg kg/day,^19^ and the European Union has set a no-observed adverse-effect level (NOAEL) of 5 mg kg/day.

The metabolism of BPA has been extensively studied using in vivo and in vitro systems. The first-pass metabolism of ingested BPA occurs in the intestine and/or liver, which greatly limits its systemic bioavailability.^20,21^ In rats, BPA is metabolized to DNA-reactive bisphenol-*o*-quinone through 5-hydroxybisphenol and bisphenol semiquinone.^22^ A study comparing the BPA-metabolizing activity of 11 forms of human hepatic cytochromes P450 (CYPs) showed that BPA is mainly metabolized by the CYP2C subfamily in human liver, and it inhibits human steroidogenic CYP17 activities.^23^ BPA is also conjugated with glucuronic acid by rat hepatic microsomes.^24^ BPA is able to extensively form BPA-monoglucuronide in rat hepatocytes or perfused liver, which is consistent with the in vivo finding that BPA-glucuronide is the major metabolite found in urine.^20,25,26^ Indeed, BPA-glucuronide is also the major metabolite in human hepatocytes, although minor amounts of BPA sulfate and the diconjugate are also present in human hepatocytes.^27^ Thus, BPA conjugation could be a determinant of its in vivo estrogenic effects, because the monoglucuronide lacks estrogenic activity.^6^ In a study to determine the extent of monoglucuronide formation in monolayer cultures of hepatocytes from rats, mice, and humans, the initial rates of metabolism in hepatocytes followed the order of mice > rats > humans.^27^ However, when extrapolated to the whole liver, the hepatic capacity for BPA glucuronidation is predicated to be humans > rats > mice, suggesting that first-pass metabolism and rapid elimination of BPA are probable following oral exposure.^27^

Fetuses can also be exposed to BPA, because it can freely cross the placenta and then bind to α-fetoprotein, resulting in enhanced bioavailability during neonatal development.^28,29^ This exposure puts fetuses and young children at risk for changes in secondary sexual characteristics as well as neural, behavioral, and immune disorders.

## ENDOCRINE-DISRUPTING EFFECTS OF BPA

The estrogenic activity of BPA represents the major endocrine-disrupting effect of BPA and was revealed in 1993 by a study showing that BPA can be released from polycarbonate flasks during autoclaving.^30^ BPA is a weak agonist to estrogen receptor β, but compared to estradiol, its activity is 1000-fold less.^31^ However, this low binding affinity does not mean that the biological activity of BPA in human is negligible, because evaluation based only on in vitro binding assays may lead to underestimation. In humans and experimental animals, BPA was originally thought to disrupt the estrogen-triggered pathways by forming a transcriptional complex that can bind the estrogen responsive element (ERE).^32,33^ In this scenario, BPA normally interacts with co-activators and co-repressors. For instance, the interaction of BPA with ER-α and ER-β has been observed to have both agonist and antagonist activities on ER-α in vitro,^34^ and even at remarkably low doses, BPA can induce estrogen-like activities in cells that are similar or even stronger than those of estrogen.^35,36^ In addition to ER-α and ER-β, BPA also binds to the ER-related receptor γ (ERR-γ).^37^ The binding of BPA to ERR-γ preserves its basal constitutive activity and also protects it from deactivation by the selective estrogen receptor modulator 4-hydroxytamoxifen.^37^ Interestingly, ERR-γ has been found in high concentrations in the placenta, which may explain reports of high BPA accumulation there and associated developmental and transplacental effects.^38^ However, additional studies suggested that BPA can also activate non-classical estrogen pathways during BPA-mediated rapid responses, including: 1) binding to the plasma membrane receptor,^39^ such as in the example of BPA binding a membrane version of the ER-α on pituitary cells and provoking Ca^2+^ influx via L-type channels^40^; 2) activating the cAMP-responsive element binding protein in a calcium-dependent manner^41^; and 3) binding to a distinct domain of ERs compared to E2.^42^ However, accumulating evidence suggests that the actions of BPA on cell signaling pathways vary among cell types and a combination of a rapid mechanism and longer effects is involved in its effects on estrogen-responsive gene expression.

In addition to its estrogen-like activity, BPA also binds to other nuclear receptors, such as androgen receptor (AR). For example, BPA could compete with 5α-dihydrotestosterone (DHT) to bind to the androgen receptor.^43,44^ Several studies demonstrated the anti-androgenic activity of BPA in cell systems through the formation of an AR/BPA complex that prevents endogenous androgens from regulating androgen-dependent gene transcription.^43,45,46^ BPA and its chlorinated derivatives also were shown to bind to thyroid hormone receptor (TR).^47,48^ Interestingly, BPA is an antagonist to the TR, whereas BPA derivatives are TR agonists that promote proliferation of rat pituitary cells.^49^

## OVARIAN CANCER

Although the pathophysiology of ovarian cancer remains poorly understood, accumulating evidence suggests that sex steroids (such as estrogens) are involved in the development of ovarian cancer.^50,51^ Approximately 50% of human ovarian epithelial cancer cells express higher levels of ER than do cells of benign tumors and normal ovary.^52,53^ Both ER-α and ER-β are expressed in normal and transformed ovarian cells, with high abundance of ER-β in granulose cells and of ER-α in theca and interstitial cells.^54^ Although an association between hormone replacement therapy and ovarian cancer remains elusive,^55,56^ prospective epidemiological studies in postmenopausal women have suggested that estrogen-only replacement therapy increases the incidence and mortality of ovarian cancer.^57,58^ Estrogen taken as an oral contraceptive in the postmenopausal years may increase risk of ovarian cancer.^59,60^ It is believed that estrogen can provide a hormonal environment that facilitates tumor progression and/or may regulate proliferation and apoptosis of ovarian cells directly.^46^ In this context, BPA exposure could mimic the effects of estrogen in ovarian cells.

Indeed, a previous epidemiological study showed that BPA is present in serum, follicular fluid, fetal serum, and full-term amniotic fluid (15- to 18-fold induction compared to other fluids), suggesting accumulation of BPA in fetuses and significant exposure during the prenatal period.^61^ BPA interrupts ovarian steroidogenesis by altering the steroidogenic enzymes.^62^ However, it remains unknown whether BPA-disrupted steroidogenesis contributes to ovarian carcinogenesis. In humans, BPA exposure increases the incidence or exacerbates the clinical course of polycystic ovary syndrome.^18,63^ In rodent models, it is well documented that neonatal exposure to BPA is associated with altered ovarian morphology, an increased number of cystic ovaries, cystic endometrial hyperplasia, and reduction in the pool of primordial follicles in the rat ovary, which is associated with an increased proliferation rate likely mediated by an estrogenic pathway.^64–68^ Prenatal exposition to BPA also causes a variety of development abnormities of the ovary (eg, endometriosis, altered number of primordial developing follicles, ovarian lesions, and inhibition of meiotic progression of oocytes).^69–71^ Nevertheless, it remains unclear whether the effects of BPA on the prenatal or neonatal developing ovary can increase the overall risk for ovarian cancer in adulthood. In adult CD-1 mice, long-term BPA exposure (18 months) also induces a significant increase in cystic ovaries and cystic endometrial hyperplasia, which are premalignant and neoplastic lesions.^68^

Furthermore, BPA was shown to regulate the expression of a battery of genes in ovarian tissues, some of which are associated with oncogenic signaling or ovarian cancer development. For instance, BPA treatment is able to up-regulate Cdk4, Ccne1, cyclin D1, ER-α, IGF-1R, and Bcl2 but down-regulate p21 and Aryl-hydrocarbon receptor nuclear translocator 2 (ARNT2), resulting in cell proliferation and inhibition of apoptosis.^11,72–74^ BPA has also been shown to regulate the transforming growth factor beta (TGF-β), JAK/STAT3, MAPK/ERK, and PI3K/Akt signaling pathways and to interact with leptin to inhibit caspase-3 expression and activity.^75,76^ BPA also stimulates granulosa-lutein cells to express matrix metalloproteinase-9 (MMP-9), an extracellular matrix protein that is associated with progression of ovarian cancer.^77,78^ The mechanism of BPA's action in ovarian tissues can be estrogenic pathway-dependent or -independent. For example, BPA exposure elicits aneuploidy of both eggs and embryos, a similar phenotype to that observed in ER-β null female mice, and presumably exerts this action via regulation of ER-β pathway genes.^79^ In another study, transplacental exposure to BPA induced changes in the expression of genes associated with estrogenic activity in ovaries of Sprague-Dawley rats.^80^ However, BPA-regulated expression of CDK4, Ccne1, Bax, and Bcl2 is independent of the estrogenic pathway,^72^ whereas BPA-induced cell proliferation in estrogen-responsive ovarian cancer cells is independent of BPA-induced MAPK activation, indicating that a tissue-specific mechanism is responsible for the regulation of cell growth by BPA.^81^ Furthermore, BPA is able to cooperate with leptin to inhibit caspase-3 expression and activity in ovarian cancer cells.^74^ According to a recent report, endocrine-disrupting chemicals promote the growth of ovarian cancer cells via the ER-CXCL12-CXCR4 signaling axis.^82^

However, there is still a lack of epidemiological data identifying associations between BPA exposure and the incidence of ovarian cancer. Available evidence raises concern regarding cancer susceptibility in ovarian tissues after prenatal or postnatal BPA exposure. Additional studies are required to determine the potential effects of BPA exposure on the development of ovarian cancer in humans.

## BREAST CANCER

Estrogen and estrogen signaling pathways play pivotal roles in the development of the mammary gland and breast carcinogenesis. Specifically, both ER-α and ER-β are first expressed at embryo stage E12.5 in the mesenchyme surrounding the bud and drive mammary ductal growth during prenatal and neonatal development. Thus, the effects of BPA exposure on mammary glands and breast carcinogenesis have been studied extensively in vitro and in vivo. Multiple lines of evidence demonstrate that fetal exposure to low doses of BPA alters cell proliferation, apoptosis, and timing in the development of mammary glands, which may further predispose the mammary gland to carcinogenesis.^83^ Indeed, BPA increases ductal density and sensitivity to estrogens after BPA exposure, which is generally shown in human breast carcinogenesis.^84^ Fetal exposure to BPA results in the in situ development of mammary gland carcinoma in a rat model with significant increases in preneoplastic lesions and intraductal proliferation.^85,86^ However, the exact mechanisms by which fetal exposure to BPA is linked to adult breast cancer remain elusive. One study showed that BPA is able to induce expression of WNT-4 and receptor activator of nuclear factor kappa-B ligand (RANKL), the two key molecules of hormone function in the regulation of mammary stem cell proliferation and carcinogenesis.^87^ Overall, either disruption of the hypothalamic–pituitary–gonadal axis or direct actions on estrogen-sensitive organs by BPA may be involved in the susceptibility of mammary gland tissue to malignant transformation. The most recent study showed the effect of low-dose BPA on the early differentiation of human embryonic stem cells into mammary epithelial cells.^88^ Circulating serum xenoestrogens also affect mammographic breast density.^89^ BPA was shown to inactivate the p53 axis and to lead to the deregulation of proliferation kinetics and cell death in non-malignant human breast epithelial cells.^90^ Taken together, the results of these studies demonstrate that BPA is likely to have significant effects on mammary epithelium and carcinogenesis.

In addition to the long-lasting effects of fetal exposure to BPA, extensive studies have examined the effects of BPA on adult normal mammary gland and transformed mammary gland cells. For example, BPA treatment promoted cell proliferation and increased cell size in mammary gland sphere cultures, but inhibited apoptosis and induced chemoresistance of different ER-positive breast cancer cell lines.^73,91–97^ Direct exposure of ER-positive breast cancer cells to BPA affected multiple oncogenic signaling pathways, including: 1) vascular endothelial growth factor (VEGF) signaling, which is associated with breast tumor angiogenesis^98^; 2) the DNA repair pathway^99^; 3) ERK1/2 activation^100,101^; and 4) STAT3 signaling.^95^ Interestingly, one report showed that BPA stimulated the GPER/EGFR/ERK pathway in the ER-null breast cancer line SKBR3, indicating the contribution of an ER-independent mechanism in BPA-mediated cancer progression.^102^ A human population-based study demonstrated that increases in serum BPA levels are associated with an increase in mammographic breast density after adjustment for age, body mass index, and other potentially confounding factors.^89^ BPA also induced a profile of tumor aggressiveness in high-risk cells from breast cancer patients characterized by high histologic grade and large tumor size, resulting in decreased recurrence-free patient survival.^103^

## PROSTATE CANCER

It is well documented that steroids play a key role in the initiation and progression of prostate cancer. In addition to androgens, estrogen is involved in the etiology of prostate cancer, and the use of anti-estrogens has been recently recognized to have a therapeutic effect in prostate cancer management.^104,105^ Both ER-α and ER-β are expressed in normal prostate and prostate cancer cells. ER-α and ER-β are found primarily in stromal and differentiated epithelium, respectively.^106,107^ It has been proposed that the prostate gland is more sensitive to estrogen exposure during the critical developmental period of the embryo stage than during adulthood.^108^ Recent evidence suggests that BPA exposure does affect prostate cells and cancer development.^109,110^ For example, transient exposure of rats to low, environmentally relevant doses of BPA or estradiol during development enhances prostate gland susceptibility to adult-onset precancerous lesions and hormonal carcinogenesis.^111^

The role of BPA in prostate tissue is relatively complicated compared to that in the mammary gland or ovary. Both steroid receptors (ERs and AR) play crucial roles in the development and progression of prostate cancer. A number of studies have shown that BPA can regulate the proliferation and migration of prostate cancer cells and induce DNA adducts in prostate cancer cells.^109,112,113^ However, the underlying molecular mechanism remains unknown (eg, through BPA-ER or BPA-AR interaction). In a xenograft model of prostate cancer cells that expresses the endogenous BPA-responsive AR-T877A mutant protein, BPA enhances tumor cell proliferation after androgen deprivation and increases the prostate-specific antigen level, indicating that the AR, at least partially, mediates the effects of BPA.^114^ In another study, BPA induced a distinct gene expression signature in prostate cancer cells expressing an AR mutation.^115^ Particularly, a major action of BPA was to down-regulate ER-β. Considering that ER-β is a molecular antagonist of AR, this study linked the interaction of BPA with ER and AR to shed light on the mechanisms of the cell proliferation-promoting effects of BPA in prostate cancer cells. However, BPA also interacts with AR, inducing nuclear translocation of the tumor-derived receptor (AR-T877A) and subsequent binding to the response elements.^116^ As AR-negative prostate cancer cells fail to show growth inhibition after exposure to high doses of BPA,^117^ both ER-β and AR could mediate the actions of BPA in advanced prostate adenocarcinomas, but only the role of AR may be essential. In addition to hormone receptor-dependent mechanisms, BPA also induces alterations in the DNA methylation patterns of multiple cell signaling genes in prostate cancer cells, indicating involvement of an epigenetic mechanism.^118^ Specifically, BPA enhances the expression of the enzyme phosphodiesterase type 4 variant 4 (PDE4D4), the gene for which is associated with DNA methylation and aging.^118^ Further studies are needed to elucidate the effects of BPA exposure on the development of prostate cancer.

## CONCLUSION AND FUTURE DIRECTIONS

**FIGURE 1:**
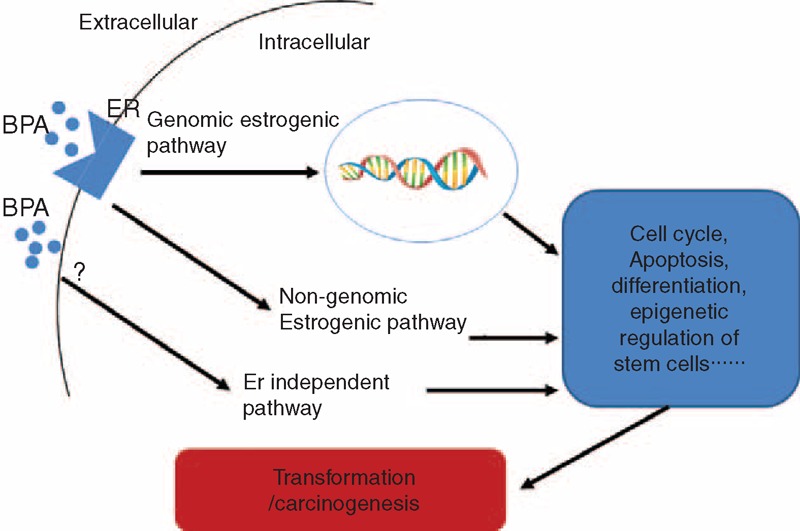
Schematic illustration of the estrogenic and estrogen-independent pathways by which BPA promotes transformation or carcinogenesis.

This review summarized the current available data regarding the role of BPA in hormone-associated cancers. Overall, several conclusions can be made: 1) BPA is a typical xenoestrogen and its estrogenic activity and estrogen-independent activity are likely responsible for its roles in promoting carcinogenesis of multiple cancers (Figure 1); 2) BPA interacts with other steroid receptor such as AR to promote proliferation of prostate cancer cells; and 3) fetal exposure to BPA could lead to “long-lasting” effects on the carcinogenesis of certain organs. Recently, one study showed increased expression of histone trimethylated H3 at lysine 27 of EZH2 after BPA treatment in human breast cancer cells, indicating epigenetic regulation by BPA of cells in carcinogenesis and progression of breast cancer.^119^ In another study, BPA treatment induced expression of microRNA-146a, which plays a role in the immune response.^120^ Thus, regulation of an epigenetic program and microRNA may represent future directions for the study of BPA's effects on hormone-related cancers, and novel results are expected to expand our understanding of the functional network of BPA in endocrine-associated cancers. A recent report demonstrated that prostate stem-progenitor cells are direct BPA targets and that exposure to BPA at low doses during development increases the hormone-dependent cancer risk in the human prostate epithelium,^121^ indicating a novel target in BPA-mediated carcinogenesis of prostate cancer. Interestingly, another study also reported that BPA exposure during puberty alters functions and gene expression in mammary stem cells, leading to early neoplasia.^122^ These two reports elucidated roles for BPA in the functions of normal or cancerous progenitor and stem cells, which represent key mechanisms of carcinogenesis. Continued characterization of the molecular and signaling mechanisms underlying the hormone-dependent and -independent effects of BPA in the growth, differentiation, and progression of cells in endocrine organs should bring a better understanding of the risks of BPA exposure in humans. Another major gap in this field is the lack of studies showing the associations of long-term BPA exposure and cancer burdens in humans, which would provide definitive evidence for evaluating the risks of BPA exposure for hormone-related cancers.

## References

[1] Brotons JA Olea-Serrano MF Villalobos M Xenoestrogens released from lacquer coatings in food cans. *Environ Health Perspect* 1995; 103:608–612. 7556016 10.1289/ehp.95103608 PMC1519121

[2] Olea N Pulgar R Perez P Estrogenicity of resin-based composites and sealants used in dentistry. *Environ Health Perspect* 1996; 104:298–305. 8919768 10.1289/ehp.96104298 PMC1469315

[3] Sajiki J Yonekubo J Leaching of bisphenol A (BPA) to seawater from polycarbonate plastic and its degradation by reactive oxygen species. *Chemosphere* 2003; 51:55–62. 12586156 10.1016/s0045-6535(02)00789-0

[4] Kawahata H Ohta H Inoue M Endocrine disrupter nonylphenol and bisphenol A contamination in Okinawa and Ishigaki Islands, Japan—within coral reefs and adjacent river mouths. *Chemosphere* 2004; 55:1519–1527. 15099732 10.1016/j.chemosphere.2004.01.032

[5] Routledge EJ White R Parker MG Differential effects of xenoestrogens on coactivator recruitment by estrogen receptor (ER) alpha and ERbeta. *J Biol Chem* 2000; 275:35986–35993. 10964929 10.1074/jbc.M006777200

[6] Matthews JB Twomey K Zacharewski TR In vitro and in vivo interactions of bisphenol A and its metabolite, bisphenol A glucuronide, with estrogen receptors alpha and beta. *Chem Res Toxicol* 2001; 14:149–157. 11258963 10.1021/tx0001833

[7] Yan PP Pan XY Wang XN [Effects of bisphenol A on the female reproductive organs and their mechanisms]. *Zhongguo Yi Xue Ke Xue Yuan Xue Bao* 2013; 35:683–688. 24382250 10.3881/j.issn.1000-503X.2013.06.018

[8] Wu HJ Liu C Duan WX Melatonin ameliorates bisphenol A-induced DNA damage in the germ cells of adult male rats. *Mutat Res* 2013; 752:57–67. 23402883 10.1016/j.mrgentox.2013.01.005

[9] Hiyama M Choi EK Wakitani S Bisphenol-A (BPA) affects reproductive formation across generations in mice. *J Vet Med Sci* 2011; 73:1211–1215. 21532259 10.1292/jvms.11-0135

[10] Brede C Fjeldal P Skjevrak I Increased migration levels of bisphenol A from polycarbonate baby bottles after dishwashing, boiling and brushing. *Food Addit Contam* 2003; 20:684–689. 12888395 10.1080/0265203031000119061

[11] Howdeshell KL Peterman PH Judy BM Bisphenol A is released from used polycarbonate animal cages into water at room temperature. *Environ Health Perspect* 2003; 111:1180–1187. 12842771 10.1289/ehp.5993 PMC1241572

[12] Kang JH Kondo F Katayama Y Human exposure to bisphenol A. *Toxicology* 2006; 226:79–89. 16860916 10.1016/j.tox.2006.06.009

[13] Richter CA Birnbaum LS Farabollini F In vivo effects of bisphenol A in laboratory rodent studies. *Reprod Toxicol* 2007; 24:199–224. 17683900 10.1016/j.reprotox.2007.06.004 PMC2151845

[14] Hanaoka T Kawamura N Hara K Urinary bisphenol A and plasma hormone concentrations in male workers exposed to bisphenol A diglycidyl ether and mixed organic solvents. *Occup Environ Med* 2002; 59:625–628. 12205237 10.1136/oem.59.9.625 PMC1740362

[15] Vandenberg LN Hauser R Marcus M Human exposure to bisphenol A (BPA). *Reprod Toxicol* 2007; 24:139–177. 17825522 10.1016/j.reprotox.2007.07.010

[16] Calafat AM Kuklenyik Z Reidy JA Urinary concentrations of bisphenol A and 4-nonylphenol in a human reference population. *Environ Health Perspect* 2005; 113:391–395. 15811827 10.1289/ehp.7534 PMC1278476

[17] Sajiki J Takahashi K Yonekubo J Sensitive method for the determination of bisphenol-A in serum using two systems of high-performance liquid chromatography. *J Chromatogr B Biomed Sci Appl* 1999; 736:255–261. 10677006 10.1016/s0378-4347(99)00471-5

[18] Takeuchi T Tsutsumi O Serum bisphenol a concentrations showed gender differences, possibly linked to androgen levels. *Biochem Biophys Res Commun* 2002; 291:76–78. 11829464 10.1006/bbrc.2002.6407

[19] Moriyama K Tagami T Akamizu T Thyroid hormone action is disrupted by bisphenol A as an antagonist. *J Clin Endocrinol Metab* 2002; 87:5185–5190. 12414890 10.1210/jc.2002-020209

[20] Pottenger LH Domoradzki JY Markham DA The relative bioavailability and metabolism of bisphenol A in rats is dependent upon the route of administration. *Toxicol Sci* 2000; 54:3–18. 10746927 10.1093/toxsci/54.1.3

[21] Upmeier A Degen GH Diel P Toxicokinetics of bisphenol A in female DA/Han rats after a single i.v. and oral administration. *Arch Toxicol* 2000; 74:431–436. 11097379 10.1007/s002040000144

[22] Atkinson A Roy D In vitro conversion of environmental estrogenic chemical bisphenol A to DNA binding metabolite(s). *Biochem Biophys Res Commun* 1995; 210:424–433. 7755618 10.1006/bbrc.1995.1678

[23] Niwa T Fujimoto M Kishimoto K Metabolism and interaction of bisphenol A in human hepatic cytochrome P450 and steroidogenic CYP17. *Biol Pharm Bull* 2001; 24:1064–1067. 11558570 10.1248/bpb.24.1064

[24] Yokota H Iwano H Endo M Glucuronidation of the environmental oestrogen bisphenol A by an isoform of UDP-glucuronosyltransferase, UGT2B1, in the rat liver. *Biochem J* 1999; 340 (Pt 2):405–409. 10333482 PMC1220264

[25] Nakagawa Y Tayama S Metabolism and cytotoxicity of bisphenol A and other bisphenols in isolated rat hepatocytes. *Arch Toxicol* 2000; 74:99–105. 10839477 10.1007/s002040050659

[26] Inoue H Yokota H Makino T Bisphenol a glucuronide, a major metabolite in rat bile after liver perfusion. *Drug Metab Dispos* 2001; 29:1084–1087. 11454725

[27] Pritchett JJ Kuester RK Sipes IG Metabolism of bisphenol a in primary cultured hepatocytes from mice, rats, and humans. *Drug Metab Dispos* 2002; 30:1180–1185. 12386122 10.1124/dmd.30.11.1180

[28] Milligan SR Khan O Nash M Competitive binding of xenobiotic oestrogens to rat alpha-fetoprotein and to sex steroid binding proteins in human and rainbow trout (Oncorhynchus mykiss) plasma. *Gen Comp Endocrinol* 1998; 112:89–95. 9748407 10.1006/gcen.1998.7146

[29] Zalko D Soto AM Dolo L Biotransformations of bisphenol A in a mammalian model: answers and new questions raised by low-dose metabolic fate studies in pregnant CD1 mice. *Environ Health Perspect* 2003; 111:309–319. 12611660 10.1289/ehp.5603 PMC1241388

[30] Krishnan AV Stathis P Permuth SF Bisphenol-A: an estrogenic substance is released from polycarbonate flasks during autoclaving. *Endocrinology* 1993; 132:2279–2286. 8504731 10.1210/endo.132.6.8504731

[31] Kuiper GG Lemmen JG Carlsson B Interaction of estrogenic chemicals and phytoestrogens with estrogen receptor beta. *Endocrinology* 1998; 139:4252–4263. 9751507 10.1210/endo.139.10.6216

[32] Kuiper GG Carlsson B Grandien K Comparison of the ligand binding specificity and transcript tissue distribution of estrogen receptors alpha and beta. *Endocrinology* 1997; 138:863–870. 9048584 10.1210/endo.138.3.4979

[33] Sheeler CQ Dudley MW Khan SA Environmental estrogens induce transcriptionally active estrogen receptor dimers in yeast: activity potentiated by the coactivator RIP140. *Environ Health Perspect* 2000; 108:97–103. 10656848 10.1289/ehp.0010897 PMC1637889

[34] Hiroi H Tsutsumi O Momoeda M Differential interactions of bisphenol A and 17beta-estradiol with estrogen receptor alpha (ERalpha) and ERbeta. *Endocr J* 1999; 46:773–778. 10724352 10.1507/endocrj.46.773

[35] Alonso-Magdalena P Morimoto S Ripoll C The estrogenic effect of bisphenol A disrupts pancreatic beta-cell function in vivo and induces insulin resistance. *Environ Health Perspect* 2006; 114:106–112. 16393666 10.1289/ehp.8451 PMC1332664

[36] Alonso-Magdalena P Laribi O Ropero AB Low doses of bisphenol A and diethylstilbestrol impair Ca2+ signals in pancreatic alpha-cells through a nonclassical membrane estrogen receptor within intact islets of Langerhans. *Environ Health Perspect* 2005; 113:969–977. 16079065 10.1289/ehp.8002 PMC1280335

[37] Matsushima A Kakuta Y Teramoto T Structural evidence for endocrine disruptor bisphenol A binding to human nuclear receptor ERR gamma. *J Biochem* 2007; 142:517–524. 17761695 10.1093/jb/mvm158

[38] Takeda Y Liu X Sumiyoshi M Placenta expressing the greatest quantity of bisphenol A receptor ERR{gamma} among the human reproductive tissues: predominant expression of type-1 ERRgamma isoform. *J Biochem* 2009; 146:113–122. 19304792 10.1093/jb/mvp049

[39] Nadal A Ropero AB Laribi O Nongenomic actions of estrogens and xenoestrogens by binding at a plasma membrane receptor unrelated to estrogen receptor alpha and estrogen receptor beta. *Proc Natl Acad Sci U S A* 2000; 97:11603–11608. 11027358 10.1073/pnas.97.21.11603 PMC17247

[40] Watson CS Bulayeva NN Wozniak AL Xenoestrogens are potent activators of nongenomic estrogenic responses. *Steroids* 2007; 72:124–134. 17174995 10.1016/j.steroids.2006.11.002 PMC1862644

[41] Quesada I Fuentes E Viso-Leon MC Low doses of the endocrine disruptor bisphenol-A and the native hormone 17beta-estradiol rapidly activate transcription factor CREB. *FASEB J* 2002; 16:1671–1673. 12207000 10.1096/fj.02-0313fje

[42] Gould JC Leonard LS Maness SC Bisphenol A interacts with the estrogen receptor alpha in a distinct manner from estradiol. *Mol Cell Endocrinol* 1998; 142:203–214. 9783916 10.1016/s0303-7207(98)00084-7

[43] Sun H Xu LC Chen JF Effect of bisphenol A, tetrachlorobisphenol A and pentachlorophenol on the transcriptional activities of androgen receptor-mediated reporter gene. *Food Chem Toxicol* 2006; 44:1916–1921. 16893599 10.1016/j.fct.2006.06.013

[44] Lee HJ Chattopadhyay S Gong EY Antiandrogenic effects of bisphenol A and nonylphenol on the function of androgen receptor. *Toxicol Sci* 2003; 75:40–46. 12805653 10.1093/toxsci/kfg150

[45] Roy P Salminen H Koskimies P Screening of some anti-androgenic endocrine disruptors using a recombinant cell-based in vitro bioassay. *J Steroid Biochem Mol Biol* 2004; 88:157–166. 15084347 10.1016/j.jsbmb.2003.11.005

[46] Xu LC Sun H Chen JF Evaluation of androgen receptor transcriptional activities of bisphenol A, octylphenol and nonylphenol in vitro. *Toxicology* 2005; 216:197–203. 16169144 10.1016/j.tox.2005.08.006

[47] Kitamura S Jinno N Ohta S Thyroid hormonal activity of the flame retardants tetrabromobisphenol A and tetrachlorobisphenol A. *Biochem Biophys Res Commun* 2002; 293:554–559. 12054637 10.1016/S0006-291X(02)00262-0

[48] Meerts IA Letcher RJ Hoving S In vitro estrogenicity of polybrominated diphenyl ethers, hydroxylated PDBEs, and polybrominated bisphenol A compounds. *Environ Health Perspect* 2001; 109:399–407. 11335189 10.1289/ehp.01109399 PMC1240281

[49] Ghisari M Bonefeld-Jorgensen EC Impact of environmental chemicals on the thyroid hormone function in pituitary rat GH3 cells. *Mol Cell Endocrinol* 2005; 244:31–41. 16221524 10.1016/j.mce.2005.01.013

[50] Cramer DW Welch WR Determinants of ovarian cancer risk. II. Inferences regarding pathogenesis. *J Natl Cancer Inst* 1983; 71:717–721. 6578367

[51] Leung PC Choi JH Endocrine signaling in ovarian surface epithelium and cancer. *Hum Reprod Update* 2007; 13:143–162. 17071638 10.1093/humupd/dml002

[52] Akahira J Suzuki T Ito K Differential expression of progesterone receptor isoforms A and B in the normal ovary, and in benign, borderline, and malignant ovarian tumors. *Jpn J Cancer Res* 2002; 93:807–815. 12149147 10.1111/j.1349-7006.2002.tb01323.x PMC5927076

[53] Lau KM Mok SC Ho SM Expression of human estrogen receptor-alpha and -beta, progesterone receptor, and androgen receptor mRNA in normal and malignant ovarian epithelial cells. *Proc Natl Acad Sci U S A* 1999; 96:5722–5727. 10318951 10.1073/pnas.96.10.5722 PMC21927

[54] Rao BR Slotman BJ Endocrine factors in common epithelial ovarian cancer. *Endocr Rev* 1991; 12:14–26. 1851084 10.1210/edrv-12-1-14

[55] Riman T Dickman PW Nilsson S Hormone replacement therapy and the risk of invasive epithelial ovarian cancer in Swedish women. *J Natl Cancer Inst* 2002; 94:497–504. 11929950 10.1093/jnci/94.7.497

[56] Coughlin SS Giustozzi A Smith SJ A meta-analysis of estrogen replacement therapy and risk of epithelial ovarian cancer. *J Clin Epidemiol* 2000; 53:367–375. 10785567 10.1016/s0895-4356(99)00179-1

[57] Rodriguez C Patel AV Calle EE Estrogen replacement therapy and ovarian cancer mortality in a large prospective study of US women. *JAMA* 2001; 285:1460–1465. 11255422 10.1001/jama.285.11.1460

[58] Lacey JV Jr Mink PJ Lubin JH Menopausal hormone replacement therapy and risk of ovarian cancer. *JAMA* 2002; 288:334–341. 12117398 10.1001/jama.288.3.334

[59] Risch HA Hormonal etiology of epithelial ovarian cancer, with a hypothesis concerning the role of androgens and progesterone. *J Natl Cancer Inst* 1998; 90:1774–1786. 9839517 10.1093/jnci/90.23.1774

[60] Garg PP Kerlikowske K Subak L Hormone replacement therapy and the risk of epithelial ovarian carcinoma: a meta-analysis. *Obstet Gynecol* 1998; 92:472–479. 9721791 10.1016/s0029-7844(98)00139-2

[61] Ikezuki Y Tsutsumi O Takai Y Determination of bisphenol A concentrations in human biological fluids reveals significant early prenatal exposure. *Hum Reprod* 2002; 17:2839–2841. 12407035 10.1093/humrep/17.11.2839

[62] Zhou W Liu J Liao L Effect of bisphenol A on steroid hormone production in rat ovarian theca-interstitial and granulosa cells. *Mol Cell Endocrinol* 2008; 283:12–18. 18191889 10.1016/j.mce.2007.10.010

[63] Diamanti-Kandarakis E Christakou C Marinakis E Phenotypes and enviromental factors: their influence in PCOS. *Curr Pharm Des* 2012; 18:270–282. 22229564 10.2174/138161212799040457

[64] Rodriguez HA Santambrosio N Santamaria CG Neonatal exposure to bisphenol A reduces the pool of primordial follicles in the rat ovary. *Reprod Toxicol* 2010; 30:550–557. 20692330 10.1016/j.reprotox.2010.07.008

[65] Kato H Ota T Furuhashi T Changes in reproductive organs of female rats treated with bisphenol A during the neonatal period. *Reprod Toxicol* 2003; 17:283–288. 12759096 10.1016/s0890-6238(03)00002-9

[66] Adewale HB Jefferson WN Newbold RR Neonatal bisphenol-a exposure alters rat reproductive development and ovarian morphology without impairing activation of gonadotropin-releasing hormone neurons. *Biol Reprod* 2009; 81:690–699. 19535786 10.1095/biolreprod.109.078261 PMC2754884

[67] Fernandez M Bourguignon N Lux-Lantos V Neonatal exposure to bisphenol a and reproductive and endocrine alterations resembling the polycystic ovarian syndrome in adult rats. *Environ Health Perspect* 2010; 118:1217–1222. 20413367 10.1289/ehp.0901257 PMC2944080

[68] Newbold RR Jefferson WN Padilla-Banks E Long-term adverse effects of neonatal exposure to bisphenol A on the murine female reproductive tract. *Reprod Toxicol* 2007; 24:253–258. 17804194 10.1016/j.reprotox.2007.07.006 PMC2043380

[69] Hunt PA Lawson C Gieske M Bisphenol A alters early oogenesis and follicle formation in the fetal ovary of the rhesus monkey. *Proc Natl Acad Sci U S A* 2012; 109:17525–17530. 23012422 10.1073/pnas.1207854109 PMC3491481

[70] Zhang HQ Zhang XF Zhang LJ Fetal exposure to bisphenol A affects the primordial follicle formation by inhibiting the meiotic progression of oocytes. *Mol Biol Rep* 2012; 39:5651–5657. 22187349 10.1007/s11033-011-1372-3

[71] Signorile PG Spugnini EP Citro G Endocrine disruptors in utero cause ovarian damages linked to endometriosis. *Front Biosci (Elite Ed)* 2012; 4:1724–1730. 22201988 10.2741/493

[72] Peretz J Craig ZR Flaws JA Bisphenol A inhibits follicle growth and induces atresia in cultured mouse antral follicles independently of the genomic estrogenic pathway. *Biol Reprod* 2012; 87:63. 22743301 10.1095/biolreprod.112.101899 PMC3464906

[73] Jung JW Park SB Lee SJ Metformin represses self-renewal of the human breast carcinoma stem cells via inhibition of estrogen receptor-mediated OCT4 expression. *PLoS ONE* 2011; 6:e28068. 22132214 10.1371/journal.pone.0028068 PMC3223228

[74] Ptak A Rak-Mardyla A Gregoraszczuk EL Cooperation of bisphenol A and leptin in inhibition of caspase-3 expression and activity in OVCAR-3 ovarian cancer cells. *Toxicol In Vitro* 2013; 27:1937–1943. 23850738 10.1016/j.tiv.2013.06.017

[75] Ptak A Gregoraszczuk EL Bisphenol A induces leptin receptor expression, creating more binding sites for leptin, and activates the JAK/Stat, MAPK/ERK and PI3K/Akt signalling pathways in human ovarian cancer cell. *Toxicol Lett* 2012; 210:332–337. 22343039 10.1016/j.toxlet.2012.02.003

[76] Park MA Choi KC Effects of 4-nonylphenol and bisphenol A on stimulation of cell growth via disruption of the transforming growth factor-beta signaling pathway in ovarian cancer models. *Chem Res Toxicol* 2014; 27:119–128. 24308608 10.1021/tx400365z

[77] Dominguez MA Petre MA Neal MS Bisphenol A concentration-dependently increases human granulosa-lutein cell matrix metalloproteinase-9 (MMP-9) enzyme output. *Reprod Toxicol* 2008; 25:420–425. 18585891 10.1016/j.reprotox.2008.05.059

[78] Schmalfeldt B Prechtel D Harting K Increased expression of matrix metalloproteinases (MMP)-2, MMP-9, and the urokinase-type plasminogen activator is associated with progression from benign to advanced ovarian cancer. *Clin Cancer Res* 2001; 7:2396–2404. 11489818

[79] Susiarjo M Hassold TJ Freeman E exposure in utero disrupts early oogenesis in the mouse. *PLoS Genet* 2007; 3:e5. 17222059 10.1371/journal.pgen.0030005 PMC1781485

[80] Naciff JM Jump ML Torontali SM Gene expression profile induced by 17alpha-ethynyl estradiol, bisphenol A, and genistein in the developing female reproductive system of the rat. *Toxicol Sci* 2002; 68:184–199. 12075121 10.1093/toxsci/68.1.184

[81] Park SH Kim KY An BS Cell growth of ovarian cancer cells is stimulated by xenoestrogens through an estrogen-dependent pathway, but their stimulation of cell growth appears not to be involved in the activation of the mitogen-activated protein kinases ERK-1 and p38. *J Reprod Dev* 2009; 55:23–29. 18854640 10.1262/jrd.20094

[82] Hall JM Korach KS Endocrine disrupting chemicals promote the growth of ovarian cancer cells via the ER-CXCL12-CXCR4 signaling axis. *Mol Carcinog* 2013; 52:715–725. 22549810 10.1002/mc.21913 PMC4287997

[83] Wang J Jenkins S Lamartiniere CA Cell proliferation and apoptosis in rat mammary glands following combinational exposure to bisphenol A and genistein. *BMC Cancer* 2014; 14:379. 24884420 10.1186/1471-2407-14-379 PMC4049406

[84] Pike MC Spicer DV Dahmoush L Estrogens, progestogens, normal breast cell proliferation, and breast cancer risk. *Epidemiol Rev* 1993; 15:17–35. 8405201 10.1093/oxfordjournals.epirev.a036102

[85] Singh M McGinley JN Thompson HJ A comparison of the histopathology of premalignant and malignant mammary gland lesions induced in sexually immature rats with those occurring in the human. *Lab Invest* 2000; 80:221–231. 10701691 10.1038/labinvest.3780025

[86] Murray TJ Maffini MV Ucci AA Induction of mammary gland ductal hyperplasias and carcinoma in situ following fetal bisphenol A exposure. *Reprod Toxicol* 2007; 23:383–390. 17123778 10.1016/j.reprotox.2006.10.002 PMC1987322

[87] Ayyanan A Laribi O Schuepbach-Mallepell S Perinatal exposure to bisphenol a increases adult mammary gland progesterone response and cell number. *Mol Endocrinol* 2011; 25:1915–1923. 21903720 10.1210/me.2011-1129 PMC5417179

[88] Yang L Luo L Ji W Effect of low dose bisphenol A on the early differentiation of human embryonic stem cells into mammary epithelial cells. *Toxicol Lett* 2013; 218:187–193. 23391485 10.1016/j.toxlet.2013.01.026

[89] Sprague BL Trentham-Dietz A Hedman CJ Circulating serum xenoestrogens and mammographic breast density. *Breast Cancer Res* 2013; 15:R45. 23710608 10.1186/bcr3432 PMC4053153

[90] Dairkee SH Luciani-Torres MG Moore DH III Bisphenol-A-induced inactivation of the p53 axis underlying deregulation of proliferation kinetics, and cell death in non-malignant human breast epithelial cells. *Carcinogenesis* 2013; 34:703–712. 23222814 10.1093/carcin/bgs379 PMC3581603

[91] Lapensee EW Tuttle TR Fox SR Bisphenol A at low nanomolar doses confers chemoresistance in estrogen receptor-alpha-positive and -negative breast cancer cells. *Environ Health Perspect* 2009; 117:175–180. 19270784 10.1289/ehp.11788 PMC2649216

[92] Miyakoshi T Miyajima K Takekoshi S The influence of endocrine disrupting chemicals on the proliferation of ERalpha knockdown-human breast cancer cell line MCF-7; new attempts by RNAi technology. *Acta Histochem Cytochem* 2009; 42:23–28. 19492024 10.1267/ahc.08036 PMC2685020

[93] Yu ZL Zhang LS Wu DS [Effects of environmental estrogens on apoptosis induced by estrogen depletion in T47D cells]. *Zhonghua Yu Fang Yi Xue Za Zhi* 2003; 37:395–397. 14703490

[94] Weber Lozada K Keri RA Bisphenol A increases mammary cancer risk in two distinct mouse models of breast cancer. *Biol Reprod* 2011; 85:490–497. 21636739 10.1095/biolreprod.110.090431 PMC3159535

[95] Zhang W Fang Y Shi X Effect of bisphenol A on the EGFR-STAT3 pathway in MCF-7 breast cancer cells. *Mol Med Rep* 2012; 5:41–47. 21909620 10.3892/mmr.2011.583

[96] Sengupta S Obiorah I Maximov PY Molecular mechanism of action of bisphenol and bisphenol A mediated by oestrogen receptor alpha in growth and apoptosis of breast cancer cells. *Br J Pharmacol* 2013; 169:167–178. 23373633 10.1111/bph.12122 PMC3632247

[97] Li X Xie W Xie C Curcumin Modulates miR-19/PTEN/AKT/p53 Axis to Suppress Bisphenol A-induced MCF-7 Breast Cancer Cell Proliferation. *Phytother Res* 2014. 10.1002/ptr.5167 24831732

[98] Buteau-Lozano H Velasco G Cristofari M Xenoestrogens modulate vascular endothelial growth factor secretion in breast cancer cells through an estrogen receptor-dependent mechanism. *J Endocrinol* 2008; 196:399–412. 18252963 10.1677/JOE-07-0198

[99] Fernandez SV Huang Y Snider KE Expression and DNA methylation changes in human breast epithelial cells after bisphenol A exposure. *Int J Oncol* 2012; 41:369–377. 22576693 10.3892/ijo.2012.1444 PMC3466112

[100] Dong S Terasaka S Kiyama R Bisphenol A induces a rapid activation of Erk1/2 through GPR30 in human breast cancer cells. *Environ Pollut* 2011; 159:212–218. 20875696 10.1016/j.envpol.2010.09.004

[101] Li M Guo J Gao W Bisphenol AF-induced endogenous transcription is mediated by ERalpha and ERK1/2 activation in human breast cancer cells. *PLoS ONE* 2014; 9:e94725. 24727858 10.1371/journal.pone.0094725 PMC3984236

[102] Pupo M Pisano A Lappano R Bisphenol A induces gene expression changes and proliferative effects through GPER in breast cancer cells and cancer-associated fibroblasts. *Environ Health Perspect* 2012; 120:1177–1182. 22552965 10.1289/ehp.1104526 PMC3440081

[103] Dairkee SH Seok J Champion S Bisphenol A induces a profile of tumor aggressiveness in high-risk cells from breast cancer patients. *Cancer Res* 2008; 68:2076–2080. 18381411 10.1158/0008-5472.CAN-07-6526

[104] Prins GS Korach KS The role of estrogens and estrogen receptors in normal prostate growth and disease. *Steroids* 2008; 73:233–244. 18093629 10.1016/j.steroids.2007.10.013 PMC2262439

[105] Smith MR Malkowicz SB Chu F Toremifene increases bone mineral density in men receiving androgen deprivation therapy for prostate cancer: interim analysis of a multicenter phase 3 clinical study. *J Urol* 2008; 179:152–155. 18001802 10.1016/j.juro.2007.08.137 PMC3090686

[106] Prins GS Marmer M Woodham C Estrogen receptor-beta messenger ribonucleic acid ontogeny in the prostate of normal and neonatally estrogenized rats. *Endocrinology* 1998; 139:874–883. 9492016 10.1210/endo.139.3.5827

[107] Prins GS Birch L Neonatal estrogen exposure up-regulates estrogen receptor expression in the developing and adult rat prostate lobes. *Endocrinology* 1997; 138:1801–1809. 9112371 10.1210/endo.138.5.5106

[108] Prins GS Birch L Tang WY Developmental estrogen exposures predispose to prostate carcinogenesis with aging. *Reprod Toxicol* 2007; 23:374–382. 17123779 10.1016/j.reprotox.2006.10.001 PMC1927084

[109] Derouiche S Warnier M Mariot P Bisphenol A stimulates human prostate cancer cell migration remodelling of calcium signalling. *Springerplus* 2013; 2:54. 23450760 10.1186/2193-1801-2-54 PMC3581770

[110] Castro B Sanchez P Torres JM exposure during adulthood alters expression of aromatase and 5alpha-reductase isozymes in rat prostate. *PLoS ONE* 2013; 8:e55905. 23405234 10.1371/journal.pone.0055905 PMC3566099

[111] Prins GS Tang WY Belmonte J Developmental exposure to bisphenol A increases prostate cancer susceptibility in adult rats: epigenetic mode of action is implicated. *Fertil Steril* 2008; 89:e41. 18308059 10.1016/j.fertnstert.2007.12.023 PMC2531072

[112] De Flora S Micale RT La Maestra S Upregulation of clusterin in prostate and DNA damage in spermatozoa from bisphenol A-treated rats and formation of DNA adducts in cultured human prostatic cells. *Toxicol Sci* 2011; 122:45–51. 21536718 10.1093/toxsci/kfr096

[113] Hess-Wilson JK Bisphenol A may reduce the efficacy of androgen deprivation therapy in prostate cancer. *Cancer Causes Control* 2009; 20:1029–1037. 19370395 10.1007/s10552-009-9337-8

[114] Wetherill YB Hess-Wilson JK Comstock CE Bisphenol A facilitates bypass of androgen ablation therapy in prostate cancer. *Mol Cancer Ther* 2006; 5:3181–3190. 17172422 10.1158/1535-7163.MCT-06-0272

[115] Hess-Wilson JK Webb SL Daly HK Unique bisphenol A transcriptome in prostate cancer: novel effects on ERbeta expression that correspond to androgen receptor mutation status. *Environ Health Perspect* 2007; 115:1646–1653. 18007998 10.1289/ehp.10283 PMC2072856

[116] Wetherill YB Petre CE Monk KR The xenoestrogen bisphenol A induces inappropriate androgen receptor activation and mitogenesis in prostatic adenocarcinoma cells. *Mol Cancer Ther* 2002; 1:515–524. 12479269

[117] Wetherill YB Fisher NL Staubach A Xenoestrogen action in prostate cancer: pleiotropic effects dependent on androgen receptor status. *Cancer Res* 2005; 65:54–65. 15665279

[118] Ho SM Tang WY Belmonte de Frausto J Developmental exposure to estradiol and bisphenol A increases susceptibility to prostate carcinogenesis and epigenetically regulates phosphodiesterase type 4 variant 4. *Cancer Res* 2006; 66:5624–5632. 16740699 10.1158/0008-5472.CAN-06-0516 PMC2276876

[119] Doherty LF Bromer JG Zhou Y In utero exposure to diethylstilbestrol (DES) or bisphenol-A (BPA) increases EZH2 expression in the mammary gland: an epigenetic mechanism linking endocrine disruptors to breast cancer. *Horm Cancer* 2010; 1:146–155. 21761357 10.1007/s12672-010-0015-9 PMC3140020

[120] Singh S Li SS Epigenetic effects of environmental chemicals bisphenol a and phthalates. *Int J Mol Sci* 2012; 13:10143–10153. 22949852 10.3390/ijms130810143 PMC3431850

[121] Prins GS Hu WY Shi GB Bisphenol A promotes human prostate stem-progenitor cell self-renewal and increases in vivo carcinogenesis in human prostate epithelium. *Endocrinology* 2014; 155:805–817. 24424067 10.1210/en.2013-1955 PMC3929731

[122] Wang D Gao H Bandyopadhyay A Pubertal bisphenol A exposure alters murine mammary stem cell function leading to early neoplasia in regenerated glands. *Cancer Prev Res (Phila)* 2014; 7:445–455. 24520039 10.1158/1940-6207.CAPR-13-0260 PMC3976434

